# Surveillance and Correlation of Antimicrobial Usage and Resistance of *Pseudomonas aeruginosa*: A Hospital Population-Based Study

**DOI:** 10.1371/journal.pone.0078604

**Published:** 2013-11-08

**Authors:** Jiancheng Xu, Xiumei Duan, Hui Wu, Qi Zhou

**Affiliations:** 1 Department of Laboratory Medicine, First Hospital of Jilin University, Changchun, China; 2 Department of Pathology, First Hospital of Jilin University, Changchun, China; 3 Department of Pediatrics, First Hospital of Jilin University, Changchun, China; Amphia Ziekenhuis, The Netherlands

## Abstract

This retrospective study evaluated trends and association between resistance of *Pseudomonas aeruginosa* isolated from patients with hospital-acquired infections (HAIs) and hospital antimicrobial usage from 2003 through 2011 in a tertiary care hospital in northeast China. HAI was defined as occurrence of infection after hospital admission, without evidence that infection was present or incubating (≦48 h) on admission. *In vitro* susceptibilities were determined by disk diffusion test and susceptibility profiles were determined using zone diameter interpretive criteria, as recommended by Clinical and Laboratory Standards Institute (CLSI). Data on usage of various antimicrobial agents, expressed as defined daily dose (DDD) per 1,000 patients-days developed by WHO Anatomical Therapeutical Chemical (ATC)/DDD index 2011, were collected from hospital pharmacy computer database. Most of 747 strains of *P. aeruginosa* were collected from respiratory samples (201 isolates, 26.9%), blood (179, 24.0%), secretions and pus (145, 19.4%) over the years. Time series analysis demonstrated a significant increase in resistance rates of *P. aeruginosa* to ticarcillin/clavulanic acid, piperacillin/tazobactam, cefoperazone/sulbactam, piperacillin, imipenem, meropenem, ceftazidime, cefepime, ciprofloxacin, and levofloxacin except aminoglycosides over time in the hospital (*P*<0.001). The rates of carbapenem-resistant *P. aeruginosa* (CRPA) isolated from patients with HAIs were 14.3%, 17.1%, 21.1%, 24.6%, 37.0%, 48.8%, 56.4%, 51.2%, and 54.1% over time. A significant increase in usage of anti-pseudomonal carbapenems (*P*<0.001) was seen. ARIMA models demonstrated that anti-pseudomonal carbapenems usage was strongly correlated with the prevalence of imipenem and meropenem-resistant *P. aeruginosa* (*P*<0.001). Increasing of quarterly CRPA was strongly correlated at one time lag with quarterly use of anti-pseudomonal carbapenems (*P*<0.001). Our data demonstrated positive correlation between anti-pseudomonal antimicrobial usage and *P. aeruginosa* resistance to several classes of antibiotics, but not all antimicrobial agents in the hospital.

## Introduction

Hospital-acquired infections (HAIs) are a significant cause of morbidity and mortality worldwide, primarily among immunocompromised and elderly people, especially if the causative organism has developed resistance to a number of antimicrobial agents. *Pseudomonas aeruginosa*, one of the main Gram-negative bacilli that cause nosocomial infections, is known for its ability to propagate on medical devices, hospital environment and even in disinfectants. Infections due to this virulent organism are difficult to both control and treat because of intrinsic resistance to a variety of antimicrobial agents [1]. It can also become resistant to multiple classes of antimicrobial agents by acquiring further resistance mechanisms, even during anti-pseudomonal chemotherapy [2]. The trend in increased antimicrobial resistance among bacterial pathogens severely limits the choice of effective antimicrobial agents. Carbapenems, including imipenem and meropenem, are β-lactam antibiotics used extensively for the treatment of *P. aeruginosa* infections. However, carbapenem-resistant *P. aeruginosa* (CRPA) has become common worldwide [3], [4]. Due to treatment failure, drug-resistant strains have been associated with higher mortality, as well as prolonged length of hospital stay and increased hospital costs compared with susceptible ones [5] [6]. The major risk factors for spread of multidrug-resistant organisms (MDROs) are poor adherence to infection control measures and overuse of certain antimicrobials [7].

The misuse and overuse of antibiotics is widespread not only in developing countries but also in the developed world. The emergence and spread of antimicrobial resistance is a complex problem that is driven by numerous interconnected factors such as under- or overuse of antimicrobials [8]. Some researches have demonstrated that prior antimicrobial drug exposure is a strong risk factor for colonization and infection due to a drug-resistant pathogen [9], [10]. However, other studies demonstrated that emergence of MDROs in a population was dependent on not only the direct effect of individual antimicrobial exposure but also the indirect effect due to increased bacterial resistance in others [11]. This might be because that the higher colonization pressure by MDROs is, the more likely the transmission of MDROs to other patients occurs [12]. Thus, studies that only evaluate individual exposure to antimicrobial agents may under-estimate the total effect of antimicrobial use on the acquisition of resistant bacteria because they do not consider these indirect effects from the antimicrobial exposures in other patients [13]. Ecological studies that use aggregated population-level data may be more suitable for such investigations because they consider the exposure of an entire population [7].

This interaction between antibiotic usage and the development of bacterial resistance to them is of particular interest with regard to *P. aeruginosa*. However, previous studies on resistance profiles of *P. aeruginosa* and antibiotic usage have reported inconsistent results at different institutions [9], [14], [15]. Aggregate antibiotic usage at local levels should therefore be monitored so that the associations between antimicrobial usage and emerging antimicrobial resistance can be evaluated. The objectives of this study were to give an overview of trends in antibiotic usage and resistance of *P. aeruginosa* isolated from patients with HAIs in a tertiary care hospital in northeast China from 2003 through 2011.

## Materials and Methods

### Ethics Statement

This study was approved by the institutional ethics committee of the First Hospital of Jilin University. The committee waived the need for informed consents (both written and oral) from participants because this was a retrospective observational study, involved very minimal risk to the subjects, did not include intentional deception, and did not involve sensitive populations or topics; this waiver does not adversely affect the rights and welfare of the subjects.

### Hospital Setting and Definitions

First Hospital is a teaching hospital affiliated with Jilin University in northeast China. It offers both primary and tertiary referral care. The number of beds progressively increased from 921 in 2003 to 2898 in 2011.

Center for Diseases Control (CDC) criteria were used for the diagnosis of nosocomial infections. HAI was defined as occurrence of infection after hospital admission, without evidence that the infection was present or incubating (≦48 h) on admission.

### Bacterial Isolates

*P. aeruginosa* isolates were subcultured to blood agar and McConkey agar plates at this laboratory for purity check and to confirm species identification. Identification was performed using the VITEK 2 system (bioMérieux, Marcy l’Etoile, France) in microbiological laboratory of the hospital. Isolates of the same species from the same patient collected during the same in-patient stay were considered duplicate isolates, and only the first isolate was included from the analysis.

### Antimicrobial Susceptibility Testing

*In vitro* susceptibilities of *P. aeruginosa* to 12 antimicrobial agents (Oxoid) were determined by the disk diffusion method and susceptibility profiles were determined using zone diameter interpretive criteria, as recommended by the Clinical and Laboratory Standards Institute (CLSI) in 2011 (M100-S21). Breakpoints of cefoperazone/sulbactam were interpreted according to the manufacturer’s recommendations. Mueller-Hinton agar (Oxoid) was used for all susceptibility tests. The proportion of resistant isolates was calculated by dividing the number of resistant isolates of *P. aeruginosa* by the total number of the isolates tested against the corresponding antibiotic multiplied by 100. *Escherichia coli* ATCC 25922, *E. coli* ATCC 35218, *Klebsiella pneumoniae* ATCC 700603, and *P. aeruginosa* ATCC 27853 were used as quality control strains for each batch of tests. Imipenem-resistant or meropenem-resistant *P. aeruginosa* was considered as CRPA. For analysis of susceptibility rates in different year and patient groups, we used the WHONET software.

### Antimicrobial Utilization

We retrospectively obtained the antimicrobial utilization information for all patients by using the hospital pharmacy computer database. The evaluated periods were from 2003 through 2011. Defined daily dose (DDD) was developed by the World Health Organization (WHO) Anatomical Therapeutical Chemical (ATC)/DDD index 2011 to standardize the comparative usage of various drugs between themselves or between different healthcare environments for all adult wards, and is defined as the assumed average maintenance dose per day for a drug used for its main indication. The amount of the antimicrobials used was calculated as DDD per 1,000 patients-days as follows: total usage measured in DDDs/(number of days in the period of data collection × number of patients) × 1,000 [8]. The six classes of antimicrobial agents analyzed in this study were: anti-pseudomonal penicillins (including piperacillin, and ticarcillin), β-lactam/β-lactamase inhibitors with anti-pseudomonal effects (cefoperazone/sulbactam, piperacillin/tazobactam, and ticarcillin/clavulanate), anti-pseudomonal cephalosporins (ceftazidime, aztreonam, and cefepime), anti-pseudomonal carbapenems (imipenem/cilastatin, and meropenem), anti-pseudomonal fluoroquinolones (ciprofloxacin, and levofloxacin), and aminoglycosides (amikacin, tobramycin, and gentamicin), modified from suggestion by CLSI in 2011.

### Statistical Analysis

Time series analysis model was used to analyze the trends in annual antimicrobial usage and antimicrobial resistance trends of *P. aeruginosa* within the study period. Autoregressive integrated moving average (ARIMA) models with cross-correlation consideration were used to determine the relationships between the trend in antimicrobial resistance of *P. aeruginosa* and antimicrobial usage over time by taking into account one time lag (delay for observing an effect of antimicrobial use) and the autocorrelation patterns. The β value indicates the variation of dependent variables when independent variables change one unit at uniform time intervals. All analyses were performed with the Statistical Package for the Social Sciences version 18.0 (SPSS, Chicago, IL, USA). All reported *P* values were two-sided, and values of *P*<0.05 were considered statistically significant.

## Results

### Bacterial Isolates

Two thousand four hundred and ten consecutive nonduplicate isolates of *P. aeruginosa* were isolated during the nine-year study period in the hospital. Among the isolates, episodes from patients with HAIs were 747. The mean age of patients with HAIs was 65.1±16.4 years. The strains were cultured from respiratory samples (201 isolates, 26.9%), followed by blood (179, 24.0%), secretions and pus (145, 19.4%), urine (102, 13.7%), pleural fluid and abdominal fluid (68, 9.1%), and bile (52, 7.0%). Three hundred and twenty-nine strains (44.0%) were from intensive care unit (ICU). Source breakdown of *P. aeruginosa* is listed in Table S1.

### Trends of *P. aeruginosa* Isolated from Patients with HAIs in Resistance to Different Antimicrobial Agents over Time

Antimicrobial resistance trends of *P. aeruginosa* isolated from patients with HAIs during the nine-year study period are listed in Table 1. Time series analysis demonstrated a significant increase in the resistance rates of *P. aeruginosa* to ticarcillin/clavulanic acid, piperacillin/tazobactam, cefoperazone/sulbactam, piperacillin, imipenem, meropenem, ceftazidime, cefepime, ciprofloxacin, and levofloxacin during nine years in the hospital (*P*<0.001). The increase of resistance rate of *P. aeruginosa* to meropenem was the highest during the nine-year study period. The β value indicated that resistance rate of *P. aeruginosa* to meropenem increased 4.6% every year according to time series analysis. During the same period, the resistance rates to gentamicin and amikacin remained stable.

**Table 1:** Antimicrobial resistance trends of *P. aeruginosa* isolated from patients with HAIs in First Hospital of Jilin University, 2003–2011.

Antimicrobial agents	Resistance rate (%) by year									Time-series analysis model		
	2003 (*n* = 28)	2004 (*n* = 35)	2005 (*n* = 38)	2006 (*n* = 57)	2007 (*n* = 81)	2008 (*n* = 127)	2009 (*n* = 101)	2010 (*n* = 121)	2011 (*n* = 159)	β	*P*	Trend
Piperacillin	28.6	34.3	42.1	47.4	55.6	46.5	44.6	57.0	57.9	4.346	<0.001	Increasing
Ticarcillin/Clavulanic acid	60.7	62.9	65.8	68.4	72.8	70.1	73.3	74.4	78.0	1.999	<0.001	Increasing
Piperacillin/Tazobactam	17.9	25.7	34.2	36.8	50.6	46.5	48.5	50.4	54.1	4.346	<0.001	Increasing
Cefoperazone/Sulbactam	14.3	22.9	31.6	36.8	44.4	41.7	42.6	43.8	49.7	4.189	<0.001	Increasing
Imipenem	10.7	11.4	15.8	17.5	29.6	38.6	39.6	41.3	43.4	4.620	<0.001	Increasing
Meropenem	7.1	8.6	13.2	17.5	28.4	37.0	37.6	38.8	40.9	4.624	<0.001	Increasing
Ceftazidime	17.9	22.9	23.7	35.1	40.7	44.1	45.5	44.6	48.4	3.923	<0.001	Increasing
Cefepime	21.4	25.7	26.3	42.1	45.7	47.2	49.5	49.6	49.7	3.806	<0.001	Increasing
Gentamicin	64.3	65.7	65.8	64.9	63.0	57.5	59.4	68.6	73.0	0.749	0.332	Stable
Amikacin	28.6	25.0	28.9	26.3	37.0	29.9	29.7	30.6	29.6	0.488	0.106	Stable
Ciprofloxacin	32.1	34.3	36.8	40.4	43.2	40.2	43.6	51.2	52.8	2.459	<0.001	Increasing
Levofloxacin	21.4	22.9	23.7	31.6	39.5	37.0	38.6	45.5	48.4	3.517	<0.001	Increasing

The rates of imipenem-resistant or meropenem-resistant *P. aeruginosa* (CRPA) isolated from patients with HAIs were 14.3%, 17.1%, 21.1%, 24.6%, 37.0%, 48.8%, 56.4%, 51.2%, and 54.1% from 2003 through 2011.

### Association of Hospital Antimicrobial Usage and Resistance of *P. aeruginosa*

Annual usage trends of antimicrobial agents used for the treatment of infections during the nine-year study period are listed in Table 2. There were no data about the usage of anti-pseudomonal penicillins because piperacillin and ticarcillin were not be used in the hospital. A significant increase in usage was seen for anti-pseudomonal carbapenems over nine years (*P*<0.001). Use of anti-pseudomonal aminoglycosides, cephalosporins, and β-lactam/β-lactamase inhibitors with anti-pseudomonal effect remained stable over the nine-year period. Time series analysis demonstrated the annual use of anti-pseudomonal fluoroquinolones slightly decreased (*P* = 0.043), whereas the usage of anti-pseudomonal fluoroquinolones fluctuated only in recent three years (2008–2010). We found the annual usage of total antimicrobial agents remained stable over nine years.

**Table 2:** Annual usage trends of antimicrobial agents used for the treatment of infections in First Hospital of Jilin University, 2003–2011.

Antimicrobial agents	Antimicrobial usage (DDDs/1000 patients/day) by year									Time-series analysis model		
	2003	2004	2005	2006	2007	2008	2009	2010	2011	β	*P*	Trend
β-lactam/β-lactamaseinhibitors	75.2	156.5	282.4	163.5	102.3	56.7	78.3	54.4	73.7	−11.314	0.263	Stable
Cephalosporins	122.2	105.3	48.6	108.6	161.7	137.9	193.9	141.9	131.2	7.591	0.098	Stable
Carbapenems	3.2	5.1	7.7	9.1	12.1	14.3	25.2	28.2	29.8	3.526	<0.001	Increasing
Aminoglycosides	43.9	44.4	44.7	42.8	40.7	32.7	37.3	75.4	24.3	0.487	0.647	Stable
Fluoroquinolones	24.5	21.5	22.1	23.1	23.8	12.9	13.9	11.6	23.5	−1.090	0.043	Decreasing
Total antimicrobial agents	269.0	332.8	405.5	347.1	340.6	254.5	348.6	311.5	282.5	−3.950	0.463	Stable

The association between resistance rates of *P. aeruginosa* isolated from patients with HAIs and usage of antimicrobial agents of different classes from 2003 through 2011 are shown in Table 3. ARIMA models demonstrated that anti-pseudomonal carbapenems usage was strongly correlated with the prevalence of imipenem and meropenem-resistant *P. aeruginosa* (*P*<0.001). Anti-pseudomonal cephalosporins were positive correlated with the prevalence of ceftazidime and cefepime-resistant *P. aeruginosa* (*P* = 0.010, 0.007, respectively). The increase of resistance rate of *P. aeruginosa* to meropenem with anti-pseudomonal carbapenems usage was the highest during the nine-year study period. The β value indicated that resistance rate of *P. aeruginosa* to meropenem increased 1.2% when anti-pseudomonal carbapenems usage increased 1 DDDs/1000 patients/day. However, we found no correlation between the usage of anti-pseudomonal β-lactam/β-lactamase inhibitors, aminoglycosides, and fluoroquinolones and the prevalence of resistant *P. aeruginosa*.

**Table 3:** Correlation between resistance rates of *P. aeruginosa* isolated from patients with HAIs and usage of antimicrobial agents in First Hospital of Jilin University, 2003–2011.

Antimicrobial agents	Time-series analysis model		
	β	*P*	Correlation
Ticarcillin/Clavulanic acid	−0.033	0.149	Negative
Piperacillin/Tazobactam	−0.066	0.209	Negative
Cefoperazone/Sulbactam	−0.048	0.331	Negative
Imipenem	1.238	<0.001	Positive
Meropenem	1.241	<0.001	Positive
Ceftazidime	0.186	0.010	Positive
Cefepime	0.195	0.007	Positive
Gentamicin	0.053	0.626	Negative
Amikacin	0.002	0.976	Negative
Ciprofloxacin	−0.477	0.252	Negative
Levofloxacin	−0.800	0.163	Negative

**Figure 1:**
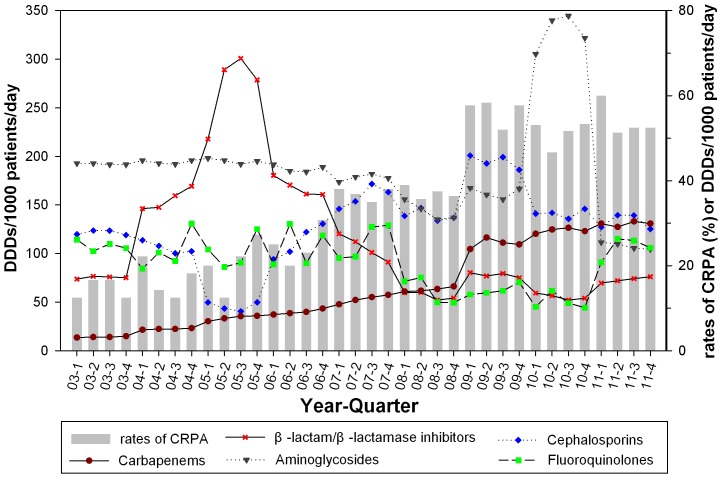
Correlation between quarterly usage of antimicrobial agents and rates of CRPA in First Hospital of Jilin University, 2003–2011. Consumption of β-lactam/β-lactamase inhibitors, and cephalosporins is expressed as defined daily dose (DDD) per 1,000 patients-days (DDDs/1000 patients/day, left y-axis). Consumption of carbapenems, aminoglycosides, and fluoroquinolones is represented as DDDs/1000 patients/day (right y-axis). The rate of CRPA is calculated by dividing the number of CRPA by the total number of the isolates multiplied by 100 (%, right y-axis). ARIMA models demonstrated that quarterly CRPA was strongly correlated at one time lag with quarterly use of anti-pseudomonal carbapenems. ARIMA models with cross-correlation consideration were used to determine the relationships between the rates of CRPA and quarterly antimicrobial usage over time by taking into account one time lag (delay for observing an effect of antimicrobial use).

Correlation between quarterly usage of antimicrobial agents and rates of CRPA isolated from patients with HAIs in the hospital from 2003 through 2011 are shown in Figure 1. ARIMA models demonstrated that increasing of quarterly CRPA was strongly correlated at one time lag with quarterly use of anti-pseudomonal carbapenems (β = 1.097, *P*<0.001); however, increasing in quarterly CRPA was associated with none of the other classes of antimicrobial agents.

## Discussion

The burden of HAIs due to MDROs may vary widely according to geographical region, healthcare setting, type of pathogen and antimicrobial substance. *P. aeruginosa* is an opportunistic pathogen responsible for a large spectrum of invasive diseases in healthcare settings, including pneumonia, urinary tract infections and bacteremia [16]. In humans, *P. aeruginosa* has been isolated from all culturable sites. The most common specimen types from patients with HAIs in the present study were respiratory samples, blood, secretions and pus over the years in this hospital.

### Resistance of *P. aeruginos*a Isolated from Patients with HAIs over Time

One aim of this study was to evaluate the resistance trend of *P. aeruginosa* isolated from patients with HAIs in recent years in this hospital. The present study showed a significant increase in resistance rates of *P. aeruginosa* to ticarcillin/clavulanic acid, piperacillin/tazobactam, cefoperazone/sulbactam, piperacillin, imipenem, meropenem, ceftazidime, cefepime, ciprofloxacin, and levofloxacin over time in the hospital. The resistance rate of *P. aeruginosa* to these antibiotics increased from 2.0% to 4.6% every year according to time series analysis. The increase of resistance rate of *P. aeruginosa* to meropenem was the highest during the nine-year study period. The resistance rates of ticarcillin/clavulanic acid and gentamicin were higher than 60.0% in almost years. During the same period, the resistance rates to gentamicin and amikacin remained stable. *P. aeruginosa* possesses a considerable degree of natural resistance to many antibiotics. This intrinsic resistance is due to low permeability of its outer membrane, antibiotics such as aminoglycosides, cephalosporins, fluoroquinolones and penicillins are recently non-sensitive to *P. aeruginosa* [17]. The growing threat of antimicrobial resistance in *P. aeruginosa* results from the extraordinary capacity of this microorganism for developing resistance to almost any available antibiotic by the selection of mutations in chromosomal genes and from the increasing prevalence of transferrable resistance determinants, particularly those encoding class B carbapenemases (or metallo-β-lactamases [MBLs]) or extended-spectrum β-lactamases (ESBLs), frequently cotransferred with genes encoding aminoglycoside-modifying enzymes [18], [19]. Among the mutation-mediated resistance mechanisms, particularly noteworthy are those leading to the repression or inactivation of the carbapenem porin OprD, the hyperproduction of the chromosomal cephalosporinase AmpC, or the upregulation of one of the several efflux pumps encoded in the *P. aeruginosa* genome [20], [21]. Furthermore, the accumulation of many of these chromosomal mutations can lead to the emergence of MDR strains, which eventually may be responsible for notable outbreaks in the hospital setting [22].

The control of MDR *P. aeruginosa* infections is a public health priority worldwide [23]. This study of 747 *P. aeruginosa* isolated from patients with HAIs over the years revealed the continuous increase of antimicrobial resistance. Carbapenems such as imipenem and meropenem are the last resort of drugs for the treatment of MDR pathogens including *P. aeruginosa*. However, the incidence of carbapenem resistance in *P. aeruginosa* increased steadily in the 2000s [3], [4]. Resistance to carbapenems, which is often accompanied with resistance to multiple other agents, has increased in all parts of the world [24]. Our study revealed the rapid increase in the prevalence of CRPA isolated from patients with HAIs over the years the hospital, from 14.3% in 2003 to 54.1% in 2011. The results of the present study indicate a strong burden of CRPA in the hospital. The development, spread, and persistence of these resistance mechanisms complicates the selection of antimicrobial therapy when trying to avoid the increased selective pressures caused by the utilization of any one class of antimicrobial agents.

### Association of Hospital Antimicrobial Usage and Resistance of *P. aeruginosa*

Another endpoint of this ecological study was to evaluate the association between resistance of *Pseudomonas aeruginosa* isolated from patients with HAIs and hospital antimicrobial usage in this hospital. Use of anti-pseudomonal aminoglycosides, cephalosporins, and β-lactam/β-lactamase inhibitors with anti-pseudomonal effect remained stable over time. The use of β-lactam/β-lactamase inhibitors with anti-pseudomonal effect increased significantly from 2003 to 2005 in that the increase of ESBL resistance in *Enterobacteriaceae* has necessitated the use of β-lactam/β-lactamase inhibitors with anti-pseudomonal effect during this period in the hospital. The usage of anti-pseudomonal aminoglycosides increased significantly in 2010, maybe due to changes of antimicrobial policies in the hospital. A significant increase in usage was seen for anti-pseudomonal carbapenems over nine years in present study. Anti-pseudomonal carbapenems usage was strongly correlated with the prevalence of imipenem and meropenem-resistant *P. aeruginosa* isolated from patients with HAIs. Increasing of quarterly CRPA was strongly correlated at one time lag with quarterly use of anti-pseudomonal carbapenems. Previous studies used individual-level data to investigate the association between antibiotic exposure and acquisition of resistant *P. aeruginosa*, and few studies have investigated aggregated population-level data [25]–[27]. Studies using individual-level data would neglect the indirect, and possibly significant, effects of antibiotics exposure of people who are near the index person [7]. Our present study used hospital-wide population-level data of 747 patients. The current study is an ecological study to investigate the association between antibiotic exposure and acquisition of resistant *P. aeruginosa*.

*In vitro* and *in vivo* studies have already documented that exposure to carbapenems increases the risk for acquiring CRPA [28], [29]. For *Enterobacteriaceae*, β-lactamases such as ESBLs and plasmid and chromosomal AmpCs are the most important resistance mechanisms. Since the ESBL SHV-2 was first reported in China in the 1990s, ESBL-producing *Enterobacteriaceae* have spread rapidly, particularly after 2000. The prevalence of ESBL-producing *E. coli* strains varies across different regions of China, with the lowest incidence in Uramuq (28.5%, Northwest), and the highest in Wuhan (78%, Central-South) in 2008 [30]. The increased importance of ESBL resistance in *Enterobacteriaceae* has necessitated the use of ESBL stable β-lactams like carbapenems in China. This followed an outbreak of CRPA infections due to use of carbapenems increased substantially in China. We found that prior exposure of imipenem and meropenem was associated with CRPA acquisition in this study. Imipenem and meropenem are broad-spectrum antibiotics with activities against most Gram-negative bacteria, including many nonfermentative Gram-negative bacilli. Therefore, it is understandable that carbapenems usage could change the bacterial flora in patients and facilitate the colonization and/or infection of resistant bacteria, such as CRPA.

We found no correlation between the usage of anti-pseudomonal β-lactam/β-lactamase inhibitors, aminoglycosides, and fluoroquinolones and the prevalence of resistant *P. aeruginosa*. There are several possible explanations for the lack of significant correlation between hospital antimicrobial usage and resistance in our study. As had previously been pointed out, resistance selection pressure occurs at the individual level and calculating antibiotic prescription using DDD measurements does not measure individual exposure to antibiotics [28], [29]. A minority of patients is exposed to the majority of broadspectrum antibiotic prescriptions in the hospital, and these are mainly the patients who are susceptible to infections by antibiotic-resistant pathogens. Hence, although DDD measurements are useful for comparison and benchmarking, they may not correlate well with subsequent antibiotic resistance development due to the inherent biases. In our study, although hospital antimicrobial usage had fluctuated, the prescription volumes had generally remained high. It is possible that beyond a certain critical threshold of antibiotic use, antibiotic resistance becomes decoupled from prescription. However, such a threshold-if it exists-has not been defined.

For improving antibiotic usage infectious diseases specialist (IDS) developed many strategies such as national guideline, antibiotics and resistance surveillance, feedback of antimicrobial resistance ratios and prior authorization of IDS for selected antimicrobial agents. The interaction between antibiotic usage and development of resistance of *P. aeruginosa* to them is complicated in real terms. In a sense, the changes in antimicrobial use are not only the cause of changes in resistance but may also be the consequence of changes in resistance patterns.

There are several limitations of this work. First, because of the low number of *P. aeruginosa* isolates in early years, there still remains uncertainty as to the results in this research. Second, this is a retrospective study, and there is no control on the real antimicrobials’ usage by patient. Third, we did not assess other antimicrobial resistance’s risk factors, duration of hospital admission, readmission rate, causes of hospitalization, and infection control measures because of missing data. Fourth, we could not discuss the outbreak of *P. aeruginosa* in different wards because of no data of pulse field electrophoresis of *P. aeruginosa* during the long study period. Fifth, because of the nature of the surveillance, we could not determine individual level or duration of exposure to antibiotics to further correlate prescription with antibiotic resistance. A well-designed prospective study to address these five limitations may be necessary in the future to observe the association between resistance of *Pseudomonas aeruginosa* and hospital antimicrobial usage in certain hospital.

## Conclusions

In conclusion, antimicrobial resistance of *P. aeruginosa* and antimicrobials usage is significant increasing in the hospital. Our data demonstrated positive correlation between anti-pseudomonal antimicrobial usage and *P. aeruginosa* resistance to several classes of antibiotics, but not all antimicrobial agents in the hospital. The effective treatment of infections caused by *P. aeruginosa* includes prevention when possible, source control measures as necessary and prompt administration of appropriate antibacterial agents. Antibacterial de-escalation should be pursued in patients with an appropriate clinical response, especially when antibacterial susceptibilities are known. Hand hygiene and barrier nursing are important to keep the spread of infection in check. Surveillance is therefore important in providing useful information for physicians in choosing empirical antibiotics. It also helps to address specific resistant issues within a region to help identify targeted intervention measures.

## Supporting Information

Table S1 Source breakdown of *P. aeruginosa* isolated from HAIs patients in First Hospital of Jilin University, 2003–2011. Patient locations are defined as ICU and non-ICU. Specimen types are expressed as respiratory, secretions and pus, urine, blood, pleural fluid and abdominnal fluid and bile.
(DOC)
