# Supplementary material for: Surveillance and Correlation of Antimicrobial Usage and Resistance of Pseudomonas aeruginosa: A Hospital Population-Based Study
Source: PLoS One. 2013 Nov 8;8(11):e78604. doi: 10.1371/journal.pone.0078604 (PMC3826718; doi:10.1371/journal.pone.0078604)
Supplement: Table S1 — Source breakdown of P. aeruginosa isolated from HAIs patients in First Hospital of Jilin University, 2003–2011. Patient locations are defined as ICU and non-ICU. Specimen types are expressed as respiratory, secretions and pus, urine, blood, pleural fluid and abdominnal fluid and bile. (DOC) [file pone.0078604.s001.doc]

**Table S1. Source breakdown of *P. aeruginosa* isolated from patients with HAIs in First Hospital of Jilin University, 2003–2011.**

| **Strata** | **2003** | |  | **2004** | |  | **2005** | |  | **2006** | |  | **2007** | |  | **2008** | |  | **2009** | |  | **2010** | |  | **2011** | |  | **Total** | |
| --- | --- | --- | --- | --- | --- | --- | --- | --- | --- | --- | --- | --- | --- | --- | --- | --- | --- | --- | --- | --- | --- | --- | --- | --- | --- | --- | --- | --- | --- |
|  | ***n*** | **%** |  | ***n*** | **%** |  | ***n*** | **%** |  | ***n*** | **%** |  | ***n*** | **%** |  | ***n*** | **%** |  | ***n*** | **%** |  | ***n*** | **%** |  | ***n*** | **%** |  | ***n*** | **%** |
| Patient location |  |  |  |  |  |  |  |  |  |  |  |  |  |  |  |  |  |  |  |  |  |  |  |  |  |  |  |  |  |
| ICU | 13 | 46.4 |  | 15 | 41.7 |  | 15 | 38.5 |  | 21 | 43.8 |  | 30 | 48.4 |  | 60 | 49.2 |  | 54 | 47.4 |  | 61 | 43.3 |  | 60 | 38.2 |  | 329 | 44.0 |
| Non-ICU | 15 | 53.6 |  | 21 | 58.3 |  | 24 | 61.5 |  | 27 | 56.3 |  | 32 | 51.6 |  | 62 | 50.8 |  | 60 | 52.6 |  | 80 | 56.7 |  | 97 | 61.8 |  | 418 | 56.0 |
| Specimen type |  |  |  |  |  |  |  |  |  |  |  |  |  |  |  |  |  |  |  |  |  |  |  |  |  |  |  |  |  |
| Respiratory | 10 | 35.7 |  | 11 | 30.6 |  | 13 | 33.3 |  | 14 | 29.2 |  | 20 | 32.3 |  | 32 | 26.2 |  | 29 | 25.4 |  | 35 | 24.8 |  | 37 | 23.6 |  | 201 | 26.9 |
| Secretions and pus | 5 | 17.9 |  | 6 | 16.7 |  | 6 | 15.4 |  | 10 | 20.8 |  | 12 | 19.4 |  | 25 | 20.5 |  | 23 | 20.2 |  | 28 | 19.9 |  | 30 | 19.1 |  | 145 | 19.4 |
| Urine | 4 | 14.3 |  | 5 | 13.9 |  | 4 | 10.3 |  | 8 | 16.7 |  | 10 | 16.1 |  | 16 | 13.1 |  | 15 | 13.2 |  | 19 | 13.5 |  | 21 | 13.4 |  | 102 | 13.7 |
| Blood | 5 | 17.9 |  | 8 | 22.2 |  | 9 | 23.1 |  | 9 | 18.8 |  | 11 | 17.7 |  | 30 | 24.6 |  | 28 | 24.6 |  | 37 | 26.2 |  | 42 | 26.8 |  | 179 | 24.0 |
| Pleural fluid and abdominal fluid | 2 | 7.1 |  | 4 | 11.1 |  | 3 | 7.7 |  | 5 | 10.4 |  | 4 | 6.5 |  | 13 | 10.7 |  | 11 | 9.6 |  | 11 | 7.8 |  | 15 | 9.6 |  | 68 | 9.1 |
| Bile | 2 | 7.1 |  | 2 | 5.6 |  | 4 | 10.3 |  | 2 | 4.2 |  | 5 | 8.1 |  | 6 | 4.9 |  | 8 | 7.0 |  | 11 | 7.8 |  | 12 | 7.6 |  | 52 | 7.0 |
| Total | 28 |  |  | 36 |  |  | 39 |  |  | 48 |  |  | 62 |  |  | 122 |  |  | 114 |  |  | 141 |  |  | 157 |  |  | 747 |  |
